# New perspectives on catecholaminergic regulation of executive circuits: evidence for independent modulation of prefrontal functions by midbrain dopaminergic and noradrenergic neurons

**DOI:** 10.3389/fncir.2014.00053

**Published:** 2014-05-21

**Authors:** Daniel J. Chandler, Barry D. Waterhouse, Wen-Jun Gao

**Affiliations:** Department of Neurobiology and Anatomy, Drexel University College of MedicinePhiladelphia, PA, USA

**Keywords:** catecholamine, dopamine, norepinephrine, prefrontal cortex, executive function

## Abstract

Cognitive functions associated with prefrontal cortex (PFC), such as working memory and attention, are strongly influenced by catecholamine [dopamine (DA) and norepinephrine (NE)] release. Midbrain dopaminergic neurons in the ventral tegmental area and noradrenergic neurons in the locus coeruleus are major sources of DA and NE to the PFC. It is traditionally believed that DA and NE neurons are homogeneous with highly divergent axons innervating multiple terminal fields and once released, DA and NE individually or complementarily modulate the prefrontal functions and other brain regions. However, recent studies indicate that both DA and NE neurons in the mammalian brain are heterogeneous with a great degree of diversity, including their developmental lineages, molecular phenotypes, projection targets, afferent inputs, synaptic connectivity, physiological properties, and behavioral functions. These diverse characteristics could potentially endow DA and NE neurons with distinct roles in executive function, and alterations in their responses to genetic and epigenetic risk factors during development may contribute to distinct phenotypic and functional changes in disease states. In this review of recent literature, we discuss how these advances in DA and NE neurons change our thinking of catecholamine influences in cognitive functions in the brain, especially functions related to PFC. We review how the projection-target specific populations of neurons in these two systems execute their functions in both normal and abnormal conditions. Additionally, we explore what open questions remain and suggest where future research needs to move in order to provide a novel insight into the cause of neuropsychiatric disorders related to DA and NE systems.

## INTRODUCTION

The prefrontal cortex (PFC) is involved in a number of cognitive and executive functions in both primates and rodents, including working memory, sustained and flexible attention (Dalley et al., 2004; Arnsten, 2009; Bari and Robbins, 2013), and is therefore critical in guiding behavior in a complex and dynamic world. Importantly, PFC is innervated and strongly modulated by a number of anatomically and neurochemically distinct pathways. Of particular interest are the afferent fibers arising in the dopaminergic ventral tegmental area (VTA) and noradrenergic locus coeruleus (LC). The anatomical characteristics of these two catecholamine nuclei, as well as the cellular, physiological, and behavioral consequences of their activation, have been well characterized and reviewed in the past dopamine [DA – (Seamans and Yang, 2004; Bjorklund and Dunnett, 2007; Grace et al., 2007; Schultz, 2007; Bromberg-Martin et al., 2010; Ungless and Grace, 2012; Roeper, 2013), norepinephrine (NE) – (Dahlstroem and Fuxe, 1964; Morrison et al., 1978; Grzanna and Molliver, 1980; Swanson, 1982; Berridge and Waterhouse, 2003; Devilbiss and Berridge, 2006; Devilbiss et al., 2006; Arnsten, 2007; Chandler and Waterhouse, 2012; Chandler et al., 2013)]. It is important to note that these two systems vary to a degree between rodents and primates. In particular, DA fibers in the primate PFC are known to arise from both the substantia nigra and VTA (Porrino and Goldman-Rakic, 1982; Haber and Fudge, 1997). In addition, in contrast to the popular view that DA-containing fibers project selectively to PFC in primates, the heaviest cortical DA projection actually terminates in motor and premotor cortices in the primate brain, while there seems to be a preferential DA projection to frontal and temporal areas in the rat with a minimal contribution to primary sensory and motor areas (Lewis et al., 1987; Berger et al., 1991). Furthermore, the distribution of DA-containing fibers among the cortical layers differs between species such that in primates, layer I is most densely innervated throughout the majority of the cortical mantle, whereas layers I through III are most densely innervated in the rat, and that this occurs preferentially in cingulate and entorhinal cortices (Berger et al., 1991). Despite the inter-species differences in DA projections to cortex [for more detailed review, see (Berger et al., 1991; Haber and Fudge, 1997)], we will focus on recent findings describing the functional organization and neuronal diversity within VTA and LC and how these attributes relate to the execution of distinct behaviors maintained by prefrontal and non-prefrontal neural circuits. We will also consider how these two systems act synergistically within their terminal fields to mutually guide several aspects of complex behaviors. Finally, although there have been many more recent breakthroughs in understanding dopaminergic neuromodulation of prefrontal circuits, we will discuss how these advances can serve as a guide to similarly transform our thinking about the LC-PFC pathway.

## DIVERSE FUNCTIONS AND PROPERTIES OF VTA DA NEURONS

It is well established that the VTA includes both DA and non-DA neurons which project heavily to both PFC and the nucleus accumbens (NAc; Swanson, 1982; Lammel et al., 2008). This projection system has been strongly linked to normal cognitive function and motivated behavior, as well as pathological deviations in these operations such as schizophrenia, attention deficit hyperactivity disorder (ADHD) and addiction (Goldman-Rakic, 1994; Goldman-Rakic and Selemon, 1997; Volkow et al., 2011, 2012). In both rodents and primates, the actions of prefrontal cortical DA are known to vary according to an “inverted U” dose response function, such that too little or too much DA impairs PFC network functions and working memory task performance (Arnsten and Goldman-Rakic, 1998; Arnsten and Li, 2005; Robbins and Arnsten, 2009). It is also known that the firing properties of VTA dopaminergic neurons are plastic such that they are capable of remaining in a silent hyperpolarized state, maintaining irregular tonic discharge, and firing phasically in response to environmental stimuli under different behavioral conditions. However, because DA seems to execute distinct operations in different terminal fields (i.e., reward and reinforcement in NAc and enhancement of working memory in PFC), it raises the question of whether or not the cells which provide DAergic innervations to these regions are anatomically distinct from one another, and whether or not these cells can be differentially activated under different circumstances. Indeed, previous studies have provided evidence for functional specialization of mesocortical DA neurons (Bannon et al., 1982; Chiodo et al., 1984). This issue was recently further illustrated and detailed by Lammel et al. (2008) who found that in rodent, PFC and NAc are in fact innervated by distinct subsets of VTA neurons, and that these cells are physiologically and phenotypically distinct from one another. Specifically, the neurons that project to NAc were found to discharge slowly and have their firing rate suppressed by application of DA, whereas those that project to PFC discharged more rapidly and did not respond to DA application. These discharge properties can be explained by the fact that PFC projection cells lack mRNA coding for the DA D2 autoreceptor, which inhibits firing of DA neurons. Taken together these findings suggest that NAc and PFC which are engaged in unique aspects of motivated behavior receive input from anatomically distinct subsets of DA containing neurons, whose firing patterns appear to be under differential control.

**FIGURE 1:**
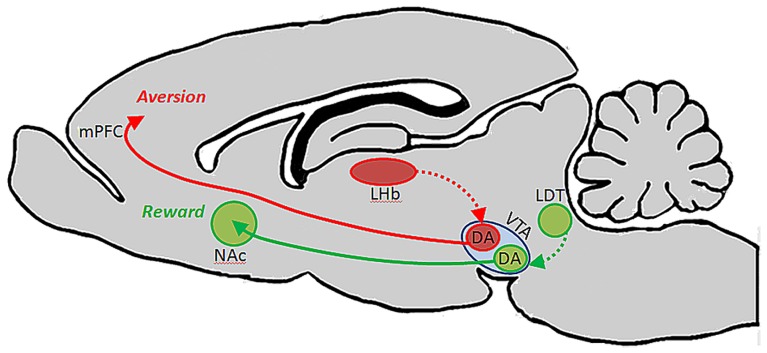
**Ventral tegmental area contains functionally heterogeneous subsets of DA neurons**. Lammel et al. (2012) showed that DA cells in VTA which receive input from laterodorsal tegmentum (LDT) selectively project to NAc, and their activation drives conditioned place preference. DA cells in VTA which receive input from lateral habenula (LHb) neurons, on the other hand, project selectively to PFC, and their activation promotes conditioned place aversion.

A follow-up to this study showed that each of these subsets of DA-containing neurons in VTA are likewise unique in their afferent regulation, and that these distinct circuits support different types of behaviors. Specifically, it was shown that afferents from the laterodorsal tegmental nucleus innervate dopaminergic VTA neurons which in turn project to NAc and elicit reward, whereas afferents from the lateral habenula (LHb) synapse on dopaminergic VTA neurons that innervate mPFC and drive aversion (**Figure 1**). The conclusion from this work is that the VTA is comprised of neurochemically similar but anatomically and functionally distinct neurons that mediate discrete aspects of motivated behaviors (Lammel et al., 2012). It is interesting to note that activation of dopaminergic neurons with projections to mPFC results in conditioned place aversion, whereas a much greater body of literature suggests that DA in the PFC plays an important role in electrophysiological and behavioral indices of working memory (Goldman-Rakic, 1994; Stevens et al., 1998; Goldman-Rakic et al., 2004; Arnsten and Li, 2005; Arnsten, 2007; Driesen et al., 2008).

These dual roles for DA in the PFC could potentially be explained by the existence of anatomically and functionally discrete subsets of VTA DA neurons that innervate different cortical layers: for example, DA neurons involved in working memory may project primarily to cortical layers that interact with primary sensory cortices to facilitate the transmission of sensory information between regions so that the representation of a stimulus can be maintained even in its absence. Aversion and emotional operations maintained by DA, on the other hand, may involve the activation of DA neurons that innervate cortical layers which maintain connections with limbic structures rather than sensory structures. In this way, activation of these two pathways could result in DA release and modulation of functionally distinct prefrontal microcircuits that mediate unique operations and behaviors. Conversely, these unique functions could be attributed to a common pool of VTA neurons that do not selectively target functionally distinct cortical layers but, depending on their pattern and level of activation, engage different receptor subtypes to elicit distinct circuit properties. For example, during modest levels of VTA output, such as in response to salient stimuli, the D2 receptor will be activitated due to its higher affinity for the transmitter. Then, during elevated levels of VTA activation, such as during periods of stress, the lower affinity D1 receptor becomes engaged. Thus, because of different receptor affinities and post-synaptic actions, DA release would produce different effects on cellular physiology and PFC circuit properties (Arnsten, 2007, 2009). Based on the inverted-U dose response function for DA actions, and the differential roles of its receptors in working memory functions, modest DA release in response to a salient stimulus is likely to strengthen measures of working memory for that stimulus, whereas excessive activation of the DA D1 receptor impairs behavioral indices of working memory (Seamans and Yang, 2004; Arnsten, 2009). During such periods when PFC is inhibited, emotional centers such as amygdala may instead take over and drive more survivalist “fight or flight” behaviors (Arnsten, 2009). In such an organization, the aversion described by Lammel et al. (2012) may have been reflective of hyperdopaminergic tone at the upper limit of the physiologic range in PFC as a result of optogenetic stimulation, thereby limiting prefrontal operations and allowing other limbic circuits to guide such a specific behavior instead. Interestingly, Bromberg-Martin et al. (2010) has hypothesized that in the primate brain, DA cells arising from substantia nigra and VTA differentially innervate orbitofrontal cortex and dorsolateral PFC to convey value and salience, respectively, to these structures. This proposal fits well with our central hypothesis that specific sub-populations of neurons arising from the midbrain and hindbrain nuclei are capable of executing unique actions in distinct terminal fields.

## DIVERSITY OF NE NEURONS IN THE LC NUCLEUS

Prefrontal circuits and operations are also subject to regulation by output from the LC-noradrenergic system. Like DA, the actions of NE vary according to an inverted-U dose response function such that too little or too much noradrenergic transmission yields a less than optimal neuronal response to sensory stimuli (Berridge and Waterhouse, 2003; Devilbiss and Waterhouse, 2004; Devilbiss et al., 2012). Importantly, the pattern of LC activation correlates highly with behavioral state in both primates and rodents such that during periods of fatigue, LC discharge is absent or slow. During periods of active waking and in conjunction with behavioral tasks that are cognitively demanding, the LC discharges faster with phasic bursts in response to relevant stimuli. During periods of stress and agitation, the nucleus discharges at a very high tonic rate and sensory-driven phasic responses are lost (Aston-Jones and Bloom, 1981a; Valentino and Foote, 1988; Aston-Jones et al., 1994; Berridge and Waterhouse, 2003; Aston-Jones and Cohen, 2005a, b). Likewise, too much NE in PFC synapses activates the α1 receptor, impairing PFC function in a manner similar to excessive activation of the D1 receptor (Arnsten and Dudley, 2005; Arnsten, 2007, 2009).

Interestingly, behavioral and electrophysiological studies of LC in both primate (Aston-Jones et al., 1994) and rodent (Bouret and Sara, 2004) have shown that LC is highly plastic in response to stimuli that drive its activation. Previous work had suggested a more simplistic role for the LC-NE system in arousal and the sleep-waking cycle. However, attended stimuli that predict reward have been found to elicit a robust phasic discharge of LC cells, while distracters of the same or different modality do not (Aston-Jones et al., 1994). Importantly, the response to a reward-predicting stimulus is rapidly lost and shifted to a new stimulus when the reward-contingency is changed (Foote et al., 1980; Aston-Jones et al., 1994; Rajkowski et al., 1994; Bouret and Sara, 2004; Aston-Jones and Cohen, 2005a). These data suggest that LC may therefore have a more complex role in attention and cognition, than simply serving as a generalized alerting or wake-promoting structure (Aston-Jones and Bloom, 1981a; Rajkowski et al., 1994; Berridge and Waterhouse, 2003; Berridge, 2008). Aston-Jones and Cohen, for example, have proposed that LC integrates goal-oriented sensory information from the PFC to shift the nucleus between tonic and stimulus-driven phasic modes of discharge. These tonic and phasic modes of discharge then sensitize terminal fields to detect non-specific and specific stimuli, respectively; thereby guiding labile versus sustained modes of attention (Aston-Jones and Cohen, 2005b).

**FIGURE 2:**
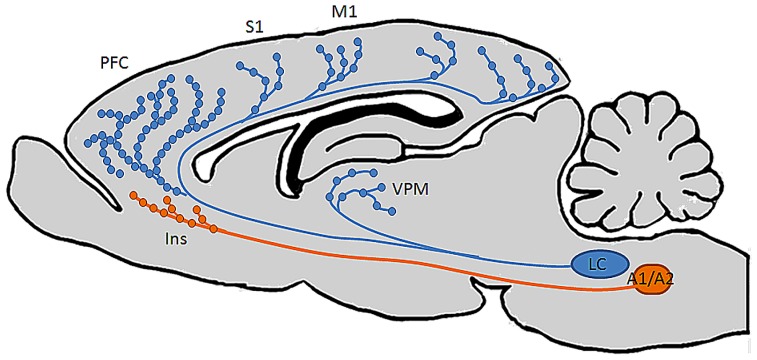
**Distinct brain regions are differentially innervated by noradrenergic neurons in multiple brainstem nuclei**. Recent findings from our laboratory (Chandler and Waterhouse, 2012; Agster et al., 2013; Chandler et al., 2013) show that individual LC neurons innervate multiple functionally distinct cortical terminal fields, and that the highest density of NE varicosities in the brain occurs in PFC. A recent finding by Robertson et al. (2013) also challenged the longstanding notion that LC is the sole source of NE to cortex by demonstrating the existence of NE-containing fibers in insular cortex derived from a rhombomere distinct from that in which LC develops, suggesting that this region has privileged access to autonomic and visceral information while the rest of the cortical mantle does not.

Importantly, it has long been thought that LC is the sole source of NE to the neocortex (Loughlin et al., 1982, 1986a, b; Berridge and Waterhouse, 2003; Agster et al., 2013), and that its’ neurons project to their terminal fields indiscriminately; i.e., a single neuron is just as likely to innervate functionally dissimilar regions as those that have common function (Loughlin et al., 1982, 1986a, b). Recent behavioral evidence however, seems to suggest that the LC-NE system exerts unique influences on operations in distinct prefrontal terminal fields. Specifically, in rodent, NE specific lesions of mPFC impair extradimensional shifting, a behavior in which animals must reorient their attentional reserves to novel stimuli to obtain food reward, but not reversal learning, an OFC dependent behavior in which animals must reorient attention to familiar but previously irrelevant stimuli (McGaughy et al., 2008b; Newman et al., 2008). On the basis of these findings and the observation that both behaviors are noradrenergically regulated (McGaughy et al., 2008a, b; Seu et al., 2009; Snyder et al., 2012) we postulated that OFC and mPFC must be innervated by distinct subsets of LC neurons: if both regions received input from a common pool of LC neurons, injection of 6-OHDA into mPFC would lead to the retrograde degeneration of the axons in mPFC, the cell bodies in LC, as well as anterograde degeneration of axon collaterals innervating OFC. Indeed, we have recently shown that these two regions, as well as anterior cingulate cortex, a third anatomically and functionally distinct prefrontal region, are in fact innervated by anatomically distinct subsets of LC neurons (Chandler and Waterhouse, 2012; Chandler et al., 2013). Additionally, another recent publication from our laboratory demonstrated that the density of noradrenergic release points is not uniform throughout the forebrain (Agster et al., 2013). Specifically, NE varicosity is significantly more dense in PFC than in sensory, motor, and thalamic regions, further supporting the hypothesis that NE may have unique roles and execute distinct operations in functionally and anatomically disparate projection fields (**Figure 2**). These findings suggest that the LC-NE projection to PFC subregions may subserve distinct behavioral roles, similar to what is suggested by the organization of the mesolimbic and mesocortical dopaminergic pathways described by Lammel et al. (2008, 2012). It has also recently been demonstrated by Robertson et al. (2013) that contrary to the long-held notion that LC is the sole source of NE-containing fibers to the forebrain in rodents, the insular cortex is innervated by non-LC derived NE terminals, i.e., sub-coeruleus, A1, and A2 cell groups (**Figure 2**). Such findings challenge the classical view that NE acts uniformly and synchronously within its terminal fields (Aston-Jones and Bloom, 1981a, b; Rajkowski et al., 1994). Specifically, NE release in insular cortex may be achieved through activation of LC, or by activation of the functionally and anatomically distinct sub-coeruleus, A1, or A2 cell groups. The different anatomical connectivities and physiological attributes of these various noradrenergic nuclei suggest that NE can be released into PFC under unique sensory or environmental circumstances. The finding that PFC is the only cortical structure in this study to be innervated by non-LC NE fibers suggests that the transmitter may maintain unique roles in prefrontal versus non-prefrontal cortical function. Because these non-LC noradrenergic cell groups receive sensory information from the viscera and are involved in homeostatic and interoceptive functions,they form an autonomic circuit and a direct route for the release of NE into prefrontal structures that affect vigilance and decision making. This pathway bypasses the LC and provides a means for asynchronous release of NE in the forebrain from multiple brainstem structures. Such an organization would therefore impose changes in prefrontal physiology without affecting properties of other terminal fields and argues that NE discretely modulates anatomically and functionally distinct terminal networks. Such a hypothesis could be tested by electrically or optogenetically stimulating these non-coerulear noradrenergic cell groups while sampling NE release in prefrontal versus non-prefrontal terminal fields by microdialysis or fast scan voltammetry.

## RECIPROCAL CONNECTIONS BETWEEN LC AND VTA

Despite the heterogeneous and varied roles for DA and NE in prefrontal cortical function that have already been discussed, an added layer of complexity emerges when taking into account the reciprocal connections maintained between VTA and LC (El Mansari et al., 2010). It is reasonable to expect that as both of these pathways are activated in response to different behavioral circumstances, each will produce some effect on the other. This then begs the question as to whether these systems work cooperatively to produce behavioral modifications that require output from both systems, or if they act competitively to drive distinct and opposite behavioral outcomes. It has been shown that electrical stimulation of LC results in an excitation followed by a brief inhibition of midbrain dopamine (DA) neurons through an α1 receptor dependent mechanism (Grenhoff et al., 1993). Furthermore, lesions of LC have been shown to reduce basal and amphetamine-induced release of DA in the NAc (Lategan et al., 1990). Interestingly, anatomical evidence has shown that there is also a monosynaptic projection from VTA to LC (Beckstead et al., 1979), and that stimulation of VTA increases the concentration of NE metabolites in PFC (Deutch et al., 1986). Furthermore, previous studies indicated that both NE and DA provide essential modulatory influences on prefrontal functions (Mingote et al., 2004; Arnsten and Li, 2005; Aston-Jones and Cohen, 2005b; Morilak et al., 2005; Rossetti and Carboni, 2005; Drouin et al., 2006).

How do these two systems coordinate their activities to appropriately regulate prefrontal functions and what happens when this coordination becomes un-balanced? Essentially, how does one system affect the ouput of the other under normal conditions and disease states? DA and NE are critical for maintaining normal, adaptive behaviors (Arnsten and Goldman-Rakic, 1998; Dalley et al., 2004; Aston-Jones and Cohen, 2005a; Arnsten, 2007; McGaughy et al., 2008a, b). Increasing or decreasing either transmitter severely limits exploratory behavior. VTA and LC neurons that release DA and NE, respectively, are both activated by salient stimuli, and the strength of activation appears to be related to the values of stimuli used for predicting future behavior (Horvitz, 2000; Stuber et al., 2008; Sara and Bouret, 2012). However, existing evidence suggests DA and NE may contribute to different functions, with DA being related to reward assessment and error prediction and NE being related to arousal and/or vigilance. This suggests that their roles in motivated behavior are segregated in that they reflect different influences of reward on behavior. It has been postulated that DA neurons are more sensitive to the incentive value of reward information, whereas NE neurons are more sensitive to the arousing aspects of reward information (Bouret et al., 2012). Similarly, during a working memory task, NE and DA systems also synergistically or complementarily contribute to modulate the persistent activity needed for the cue, delay and response signaling within the PFC circuitry. Specifically, as others (Robbins and Arnsten, 2009; Arnsten, 2009) have proposed, with optimal levels of NE or DA release under alert, non-stressed conditions, PFC neurons fire during the delay period following cues for preferred but not non-preferred directions. NE enhances delay-related firing in response to cues in preferred directions by stimulating α2A-receptors (increasing the “signal”), whereas DA weakens delay-related firing in response to cues in non-preferred directions by stimulating D1 receptors (decreasing the “noise”). This assumption is evidenced by administration of appropriate concentrations of the α2A-receptor agonist guanfacine or the D1 receptor agonist SKF81297. In contrast, with high levels of NE and DA release as would occur during stress, NE engages the lower-affinity α1-receptors and reduces mnemonic stimulus evoked neuronal firing. Interestingly, the impact of the activation of adrenergic receptors in non-prefrontal cortical regions such as sensory and motor cortices seem to be opposite of that in prefrontal regions: α1-receptor activation increasees neuronal responsiveness to sensory-driven inputs, whereas α2 receptor activation suppresses stimulus evoked discharge (Arnsten, 2000, 2007, 2009). Similarly, high DA induces excessive D1 receptor stimulation and suppresses cell firing as well. Indeed, administration of the α1-receptor agonist phenylephrine (Mao et al., 1999) or a high concentration of SKF81297 (Williams and Goldman-Rakic, 1995) can mimick the effects of high NE and DA levels, respectively.

It is also important to recognize that DA and NE levels in PFC are constantly fluctuating as a function of arousal level and ongoing behavioral contingencies. As the relative levels of these transmitters in the extracellular space changes, so too will their impact on cellular function. Importantly, the impact of these transmitter systems on post-synaptic cellular physiology is often characterized one at a time, i.e., the impact of DA or the impact of NE on specific parameters of neuronal or circuit function. However, under physiological conditions, it is likely that these two transmitters, as well as many other neuromodulatory agents and transmitter substances interact simultaneously throughout the brain and spinal cord via activation of a number of membrane-bound receptors on neurons and glia. A first step in addressing the issue of neuromodulator interactions and influences on complex circuit functions would be to consider the net effects of simultaneous administration of two or more modulatory substances on synaptically driven discharge of target neurons. There is already strong evidence for synapse- and cell-type specific modulation of local cortical circuitry in the PFC by both DA and NE (Gao et al., 2001, 2003; Gao and Goldman-Rakic, 2003; Wang et al., 2013). Thus, the PFC is a likely candidate for studies focused on the combined impact of DA and NE on transmission at single synapses and response properties of identified neurons.

## SUMMARY

**FIGURE 3:**
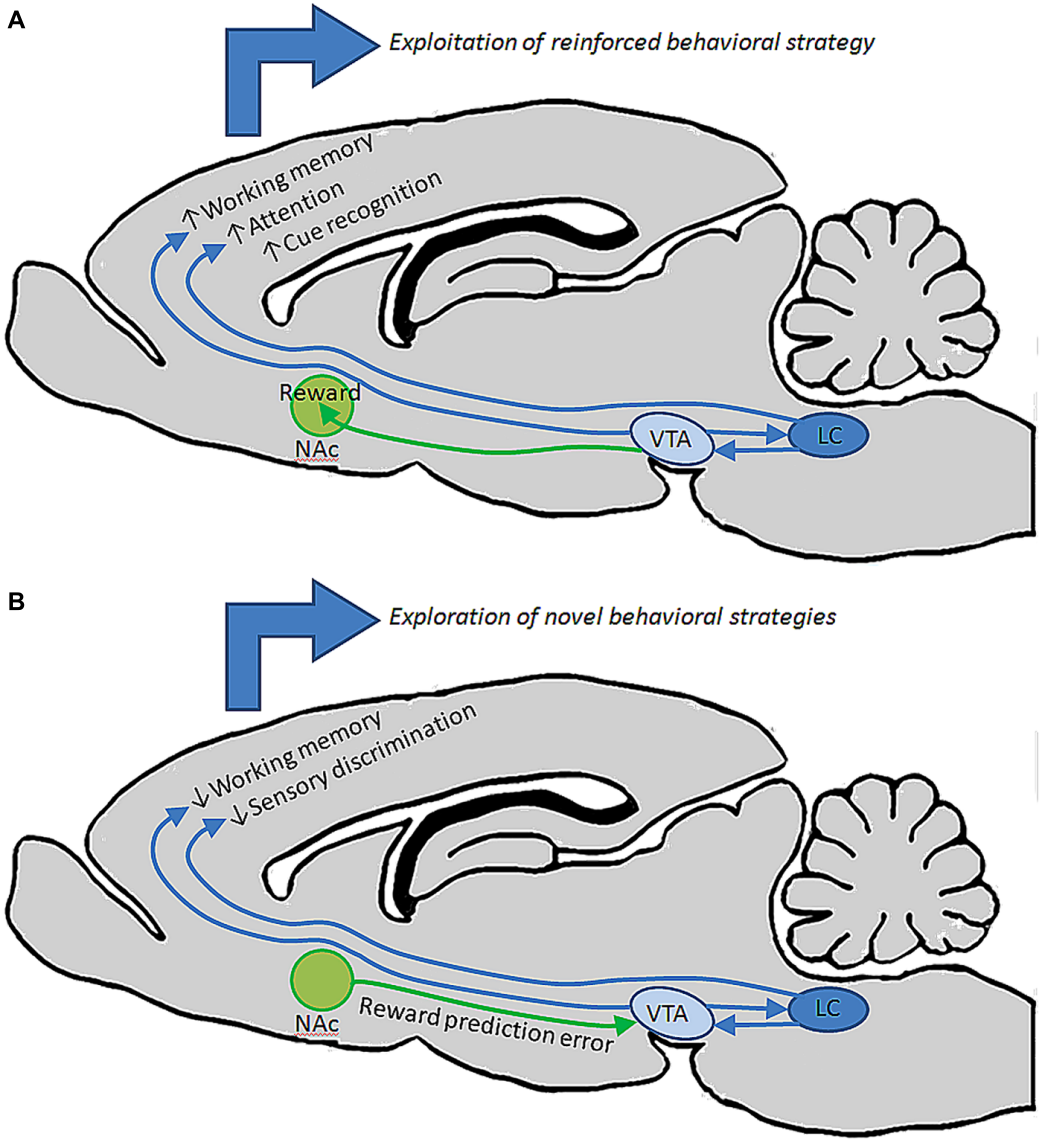
**entral tegmental area and LC may work together to guide behavior under different circumstances. (A)** During performance in a behavioral task in which an animal is successfully retrieving reward, reciprocal connections between VTA and LC may facilitate elevated output from these nuclei, driving release of DA and NE in PFC to promote working memory, attention, and discrimination of specific stimuli that predict reward. These behavioral operations could collectively contribute to the repetition of that behavior until reward is retrieved. **(B)** When a previously successful behavioral strategy loses its relevance, NAc may signal to VTA that an expected reward has not occurred. Changes in VTA output could then alter LC output, collectively changing the level of DA and NE release in PFC to diminish working memory and discrimination of specific stimuli, instead allowing the animal to explore new behavioral strategies on the basis of detecting previously irrelevant stimuli.

**FIGURE 4:**
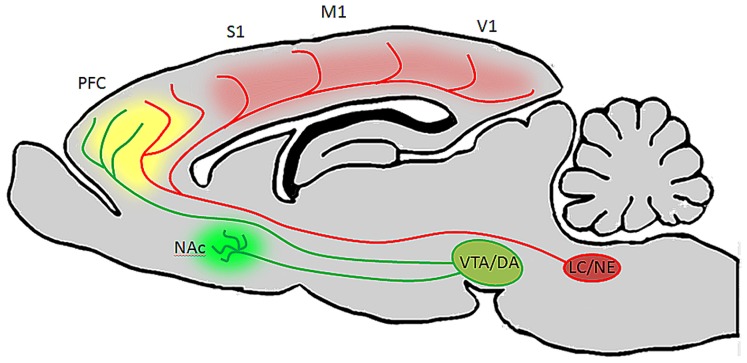
**Ventral tegmental area and LC neurons have distinct targets but their efferent fibers converge in PFC**. In the rodent brain, LC projects heavily to the entire cortical mantle, including PFC and primary sensory and motor areas, but not to the striatum or NAc (Berridge and Waterhouse, 2003). VTA on the other hand innervates NAc and PFC, but provides only sparse innervations to more posterior cortical areas (Berger et al., 1991). Therefore, during periods of arousal and vigilance, when LC and VTA discharge is elevated, DA will be released in NAc, LC will be released in posterior cortical areas, and both catecholamines will be released in PFC. This may be beneficial during behavioral tasks which require sustained attention, as DA in NAc (green) will facilitate reward, NE in cortex (red) will alter the signal to noise ratio of pyramidal neurons to optomize them to specific stimuli, and both catecholamines in PFC (yellow) will work synergistically to facilitate working memory and attention to relevant stimuli.

Taken together the findings reviewed here suggest that both noradrenergic and dopaminergic nuclei contain heterogeneous sets of neurons whose properties vary according to terminal field projection targets, and that these two catecholamine pathways act synergistically or complementarily in order to affect executive function and motivated behaviors via connections with specified forebrain circuits as well as by maintaining reciprocal excitatory connections with one another. Because there exists a range of concentrations for both DA and NE in PFC at which behavior and cellular physiology are optimized, and too far below or above this range is detrimental to behavioral outcomes, it seems that these two systems are both required for the normal maintenance and execution of prefrontal operations. Likewise, because these two pathways are reciprocally excitatory, it is likely that activation of one pathway by external or internal stimuli recruits the other indirectly. Such an arrangement would benefit complex behaviors, i.e., a task requiring sustained attention is also dependent on motivational state. It may be the case that VTA efferents to NAc and PFC work in concert with LC inputs to PFC and primary sensory and motor cortical regions. For example, during a period of vigilance in a particular behavioral task, LC activation and NE release may optimize PFC and sensory cortical function with respect to signal to noise ratios of stimulus evoked pyramidal neuron responses, while DA release from VTA promotes a transient working memory association – mnemonic – of that stimulus. Together, these two transmitter systems work synergistically to allow the animal to selectively focus on and remember the relevance of a reward associated stimulus. Upon the successful execution of a behavioral trial and reward retrieval, VTA signals NAc to elicit reward, reinforcing the behavior and causing the animal to continue focusing on that specific stimulus to predict and retrieve the next reward. Hereafter, when a behavioral contingency is changed, the NAc signals VTA that an expected reward has not occurred. The reciprocal connections between VTA and LC may then alter their collective output in PFC, thereby decreasing the sensitivity of PFC and primary sensory networks to that specific stimulus by a NE-mediated decrease in signal to noise ratio, as well as a decrease in working memory for that stimulus. Consequently, in the absence of reinforcement and reward, the animal is able to sample alternative behavioral strategies through sensitization to previously irrelevant stimuli. Once a new strategy is identified, VTA signals NAc to promote reward, thereby shifting the reciprocal connections between LC and VTA back to a mode which favors sensory discrimination and working memory of the new reward predictive stimulus (**Figure 3**). This is an intriguing possibility given that in the rodent, NAc and striatum seem to be largely devoid of LC-derived fibers, and primary sensory and motor cortical areas are not heavily innervated by DA fibers (Berger et al., 1991; Berridge and Waterhouse, 2003). Hence, VTA may preferentially modulate reward through its projections to NAc, LC may preferentially modulate sensory and motor processes through its projections to more posterior cortical areas, and these two catecholamine nuclei may work synergistally in PFC to affect attention, working memory, and cognitive functions that drive complex behavior (**Figure 4**).

Therefore, reciprocal connections between these two nuclei may be important for maintaining activity states in each nucleus that are sufficient for appropriately guiding ongoing behavior. In the absence of these reciprocal connections, the projection from VTA to NAc might be sufficient for keeping an animal motivated to perform a task or execute a specific behavior, but attention toward a specific stimulus used to guide that behavior may be minimal. Conversely, the projection from LC to PFC might be adequate in resolving specific stimuli, but insufficient to attend specific stimuli and achieve a desirable outcome in the absence of a motivational drive provided by the dopaminergic projection from VTA to NAc.

Additionally, as discussed earlier, the VTA maintains a projection to PFC which has been shown to promote aversion (Lammel et al., 2012) rather than motivation or reward. Interestingly, it is known that certain stressors elicit greater release and metabolism of DA in PFC than other forebrain regions (Deutch and Roth, 1990), suggesting that the mesocortical DA may play an integral role in the cognitive aspects of the stress response. Importantly, it is also known that high levels of DA and NE in PFC impair cognition and elevation of these catecholamines occur during exposure to stressors. During stressor-induced activation of the LC (Valentino and Foote, 1987, 1988; Curtis et al., 1999; Berridge and Waterhouse, 2003; Berridge, 2008; Devilbiss et al., 2012), the VTA would be the target of increased noradrenergic transmission from the LC-VTA pathway, thereby providing a means for VTA to contribute to the expression of aversive behaviors much in the same way that LHb neurons influence VTA activity and DA release within the PFC (Lammel et al., 2012, 2013). Methods similar to those used by Lammel et al. (2012) could be employed to identify such functional connections between VTA and LC and to determine how the reciprocal connections between these two nuclei influence physiological properties, release, and consequently PFC related cognitive function and behavior.

Importantly, these recent findings on the neurobiology of the VTA, as well as the recent identification of non-LC derived NE-containing terminals in insular cortex represent a way forward for advancing the study of the LC-NE pathway. As this system has long been viewed as homogeneous with fairly uniform, synchronous actions across its efferent domain and on behavior by way of a highly divergent network of axon collaterals, the demonstration that it is in fact more heterogeneous than previously recognized would transform the prevailing notions about the postulated contributions of the LC-NE system to forebrain operations. Importantly, we have recently provided anatomical evidence that LC neurons innervate their terminal fields on a functional rather than random basis (Chandler and Waterhouse, 2012; Chandler et al., 2013) and experiments are currently underway to test the hypothesis that cells with discrete terminal fields express different molecular profiles and unique physiological attributes. Such data would provide evidence that LC efferent system is capable of differential release and asynchronous NE actions across its terminal fields in the same way that DA release is governed by specified VTA projection patterns. Additionally, the recent demonstration that certain regions of PFC are innervated by non-LC-NE containing fibers (Robertson et al., 2013) supports the view that NE maintains distinctive roles in prefrontal circuit operations as dictated by activation of source nuclei (sub-coeruleus, A1, A2) that give rise to NE-PFC projections. Such an organization would therefore prompt noradrenergic modulatory actions in prefrontal circuits without affecting other cortical regions; a mode of operation similar to that proposed for the VTA-DA system on the basis of its divergent mesocortical and mesolimbic projections.

Identification of specific afferents to LC cells with specified outputs as has been shown in the DA system (Lammel et al., 2008, 2012) will further the collective understanding of the role of LC in maintaining discrete behavioral operations rather than acting as a homogeneous and uniform modulator of the activity in LC projection fields. Optogenetic approaches may provide a means of characterizing anatomic, neurochemical, and functionally specific pathways into and out of LC that maintain distinct roles and demonstrate that NE release is capable of producing unique actions in different terminal fields under diverse circumstances. Because the LC-NE and VTA-DA systems maintain reciprocal anatomical connections and appear to act synergistically and complementarily to guide behavior, advances in the study of one of these catecholamine pathways will by necessity impact study of the other. Going forward it will be important to consider the differences as well as the similarities between these two systems. Nevertheless, the results of recent studies of the VTA show that heterogeneity is quite apparent in the nucleus (Lammel et al., 2008, 2012), and our recent work on the anatomy of the LC-PFC projections show that the nucleus is at least anatomically aligned to allow for similar heterogeneity in this nucleus as well. As such, anatomical, molecular, and physiological heterogeneity in catecholamine nuclei may therefore be a fundamental principle of their organization, and future studies of these structures and their efferent domains may provide a framework for better understanding acquired or genetically transmitted abnormalities of the VTA-DA and LC-NE systems that result in maladaptive behaviors including those expressed in addiction, ADHD, schizophrenia, and post-traumatic stress disorder.

## FUTURE PERSPECTIVE AND FUNCTIONAL IMPLICATIONS OF THE DIVERSITIES IN CATECHOLAMINERGIC INNERVATION OF PFC

The diverse innervation of PFC by subsets of DA and NE neurons is certainly an important conceptual advance in our understanding of these two systems. But several questions remain. How are these two systems affected when PFC function and structure are altered in response to genetic and epigenetic factors? How do disease states affect each of these systems and their interactions? Are all cells within these nuclei equally responsive to genetic and environmental insult, or is it possible that cells with different terminal fields are differentially susceptible to certain forms of stressors? For example, evidence suggests that in Alzheimer’s and Parksinson’s diseases, LC neurons degenerate selectively (Gesi et al., 2000; Grimm et al., 2004; Weinshenker, 2008; Szot et al., 2010; McMillan et al., 2011; Miguelez et al., 2011). It may be that such degeneration targets LC-PFC projection neurons specifically and that this selective degeneration plays a role in the cognitive decline associated with these diseases. Further exploration of the properties of specified groups of LC-cortical projection neurons could help determine the susceptibility of these organizations to pharmacological, environmental, or genetic insult that manifest in symptoms of neuropsychiatric or neurodegenerative disease associated with noradrenergic function. Similarly, it remains to be determined whether PFC projection neurons in the LC are more sensitive to stressors or the actions of psychostimulant drugs (e.g., methylphenidate) as compared to LC cells with different efferent domains. Furthermore, based on the published data on VTA neurons, we expect that subtypes of LC neurons with unique profiles and terminal field projection patterns receive different sets of afferent inputs, e.g., GABAergic versus glutamatergic, cortical versus subcortical, as well as dopaminergic, serotoninergic, or cholinergic afferents. Answers to these questions will provide novel insights into the operation of these systems and their collective impact on adaptive and maladaptive behavior.

## Conflict of Interest Statement

The authors declare that the research was conducted in the absence of any commercial or financial relationships that could be construed as a potential conflict of interest.

## References

[B1] Agster K. L. Mejias-Aponte C. A. Clark B. D. Waterhouse B. D. (2013). Evidence for a regional specificity in the density and distribution of noradrenergic varicosities in rat cortex. *J. Comp. Neurol.* 521 2195–2207 10.1002/cne.23270 23184811 PMC4529674

[B2] Arnsten A. F. (2000). Through the looking glass: differential noradenergic modulation of prefrontal cortical function. *Neural Plast.* 7 133–146 10.1155/NP.2000.133 10709220 PMC2565372

[B3] Arnsten A. F. (2007). Catecholamine and second messenger influences on prefrontal cortical networks of “representational knowledge”: a rational bridge between genetics and the symptoms of mental illness. *Cereb. Cortex* 17(Suppl. 1) i6–i15 10.1093/cercor/bhm033 17434919

[B4] Arnsten A. F. (2009). Stress signalling pathways that impair prefrontal cortex structure and function. *Nat. Rev. Neurosci.* 10 410–422 10.1038/nrn2648 19455173 PMC2907136

[B5] Arnsten A. F. Dudley A. G. (2005). Methylphenidate improves prefrontal cortical cognitive function through alpha2 adrenoceptor and dopamine D1 receptor actions: relevance to therapeutic effects in Attention Deficit Hyperactivity Disorder. *Behav. Brain Funct.* 1:2 10.1186/1744-9081-1-2 PMC1143775 15916700

[B6] Arnsten A. F. Goldman-Rakic P. S. (1998). Noise stress impairs prefrontal cortical cognitive function in monkeys: evidence for a hyperdopaminergic mechanism. *Arch. Gen. Psychiatry* 55 362–368 10.1001/archpsyc.55.4.362 9554432

[B7] Arnsten A. F. Li B. M. (2005). Neurobiology of executive functions: catecholamine influences on prefrontal cortical functions. *Biol. Psychiatry* 57 1377–1384 10.1016/j.biopsych.2004.08.019 15950011

[B8] Aston-Jones G. Bloom F. E. (1981a). Activity of norepinephrine-containing locus coeruleus neurons in behaving rats anticipates fluctuations in the sleep-waking cycle. *J. Neurosci.* 1 876–886 7346592 10.1523/JNEUROSCI.01-08-00876.1981 PMC6564235

[B9] Aston-Jones G. Bloom F. E. (1981b). Norepinephrine-containing locus coeruleus neurons in behaving rats exhibit pronounced responses to non-noxious environmental stimuli. *J. Neurosci.* 1 887–900 7346593 10.1523/JNEUROSCI.01-08-00887.1981 PMC6564231

[B10] Aston-Jones G. Cohen J. D. (2005a). Adaptive gain and the role of the locus coeruleus-norepinephrine system in optimal performance. *J. Comp. Neurol.* 493 99–110 10.1002/cne.20723 16254995

[B11] Aston-Jones G. Cohen J. D. (2005b). An integrative theory of locus coeruleus-norepinephrine function: adaptive gain and optimal performance. *Annu. Rev. Neurosci.* 28 403–450 10.1146/annurev.neuro.28.061604.135709 16022602

[B12] Aston-Jones G. Rajkowski J. Kubiak P. Alexinsky T. (1994). Locus coeruleus neurons in monkey are selectively activated by attended cues in a vigilance task. *J. Neurosci.* 14 4467–4480 8027789 10.1523/JNEUROSCI.14-07-04467.1994 PMC6577022

[B13] Bannon M. J. Reinhard J. F. Bunney E. B. Jr. Roth R. H. (1982). Unique response to antipsychotic drugs is due to absence of terminal autoreceptors in mesocortical dopamine neurones. *Nature* 296 444–446 10.1038/296444a0 7063040

[B14] Bari A. Robbins T. W. (2013). Inhibition and impulsivity: behavioral and neural basis of response control. *Prog. Neurobiol.* 108 44–79 10.1016/j.pneurobio.2013.06.005 23856628

[B15] Beckstead R. M. Domesick V. B. Nauta W. J. (1979). Efferent connections of the substantia nigra and ventral tegmental area in the rat. *Brain Res.* 175 191–217 10.1016/0006-8993(79)91001-1 314832

[B16] Berger B. Gaspar P. Verney C. (1991). Dopaminergic innervation of the cerebral cortex: unexpected differences between rodents and primates. *Trends Neurosci*. 14 21–27 10.1016/0166-2236(91)90179-X 1709528

[B17] Berridge C. W. (2008). Noradrenergic modulation of arousal. *Brain Res. Rev.* 58 1–17 10.1016/j.brainresrev.2007.10.013 18199483 PMC2517224

[B18] Berridge C. W. Waterhouse B. D. (2003). The locus coeruleus-noradrenergic system: modulation of behavioral state and state-dependent cognitive processes. *Brain Res. Brain Res. Rev.* 42 33–84 10.1016/S0165-0173(03)00143-7 12668290

[B19] Bjorklund A. Dunnett S. B. (2007). Dopamine neuron systems in the brain: an update. *Trends Neurosci.* 30 194–202 10.1016/j.tins.2007.03.006 17408759

[B20] Bouret S. Sara S. J. (2004). Reward expectation, orientation of attention and locus coeruleus-medial frontal cortex interplay during learning. *Eur. J. Neurosci.* 20 791–802 10.1111/j.1460-9568.2004.03526.x 15255989

[B21] Bouret S. Ravel S. Richmond B. J. (2012). Complementary neural correlates of motivation in dopaminergic and noradrenergic neurons of monkeys. *Front. Behav. Neurosci.* 6:40. 10.3389/fnbeh.2012.00040 PMC3398259 22822392

[B22] Bromberg-Martin E. S. Matsumoto M. Hikosaka O. (2010). Dopamine in motivational control: rewarding, aversive, and alerting. *Neuron* 68 815–834 10.1016/j.neuron.2010.11.022 21144997 PMC3032992

[B23] Chandler D. Waterhouse B. D. (2012). Evidence for broad versus segregated projections from cholinergic and noradrenergic nuclei to functionally and anatomically discrete subregions of prefrontal cortex. *Front. Behav. Neurosci.* 6:20. 10.3389/fnbeh.2012.00020 PMC3356860 22661934

[B24] Chandler D. J. Lamperski C. S. Waterhouse B. D. (2013). Identification and distribution of projections from monoaminergic and cholinergic nuclei to functionally differentiated subregions of prefrontal cortex. *Brain Res*. 1522 38–58 10.1016/j.brainres.2013.04.057 23665053 PMC3811940

[B25] Chiodo L. A. Bannon M. J. Grace A. A. Roth R. H. Bunney B. S. (1984). Evidence for the absence of impulse-regulating somatodendritic and synthesis-modulating nerve terminal autoreceptors on subpopulations of mesocortical dopamine neurons. *Neuroscience* 12 1–16 10.1016/0306-4522(84)90133-7 6462443

[B26] Curtis A. L. Pavcovich L. A. Valentino R. J. (1999). Long-term regulation of locus ceruleus sensitivity to corticotropin-releasing factor by swim stress. *J. Pharmacol. Exp. Ther.* 289 1211–1219 10336508

[B27] Dahlstroem A. Fuxe K. (1964). Evidence for the existence of monoamine-containing neurons in the central nervous system. I. demonstration of monoamines in the cell bodies of brain stem neurons. *Acta Physiol. Scand. Suppl.* 62(Suppl. 232) 1–55 14229500

[B28] Dalley J. W. Cardinal R. N. Robbins T. W. (2004). Prefrontal executive and cognitive functions in rodents: neural and neurochemical substrates. *Neurosci. Biobehav. Rev.* 28 771–784 10.1016/j.neubiorev.2004.09.006 15555683

[B29] Deutch A. Y. Roth R. H. (1990). The determinants of stress-induced activation of the prefrontal cortical dopamine system. *Prog. Brain Res.* 85 367–402; discussion 402–403 10.1016/S0079-6123(08)62691-6 2094906

[B30] Deutch A. Y. Goldstein M. Roth R. H. (1986). Activation of the locus coeruleus induced by selective stimulation of the ventral tegmental area. *Brain Res.* 363 307–314 10.1016/0006-8993(86)91016-4 3942901

[B31] Devilbiss D. M. Berridge C. W. (2006). Low-dose methylphenidate actions on tonic and phasic locus coeruleus discharge. *J. Pharmacol. Exp. Ther.* 319 1327–1335 10.1124/jpet.106.110015 16980569

[B32] Devilbiss D. M. Page M. E. Waterhouse B. D. (2006). Locus ceruleus regulates sensory encoding by neurons and networks in waking animals. *J. Neurosci.* 26 9860–9872 10.1523/JNEUROSCI.1776-06.2006 17005850 PMC6674489

[B33] Devilbiss D. M. Waterhouse B. D. (2004). The effects of tonic locus ceruleus output on sensory-evoked responses of ventral posterior medial thalamic and barrel field cortical neurons in the awake rat. *J. Neurosci.* 24 10773–10785 10.1523/JNEUROSCI.1573-04.2004 15574728 PMC6730210

[B34] Devilbiss D. M. Waterhouse B. D. Berridge C. W. Valentino R. (2012). Corticotropin-releasing factor acting at the locus coeruleus disrupts thalamic and cortical sensory-evoked responses. *Neuropsychopharmacology* 37 2020–2030 10.1038/npp.2012.50 22510725 PMC3398725

[B35] Driesen N. R. Leung H. C. Calhoun V. D. Constable R. T. Gueorguieva R. Hoffman R. (2008). Impairment of working memory maintenance and response in schizophrenia: functional magnetic resonance imaging evidence. *Biol. Psychiatry* 64 1026–1034 10.1016/j.biopsych.2008.07.029 18823880 PMC2650279

[B36] Drouin C. Page M. Waterhouse B. (2006). Methylphenidate enhances noradrenergic transmission and suppresses mid- and long-latency sensory responses in the primary somatosensory cortex of awake rats. *J. Neurophysiol.* 96 622–632 10.1152/jn.01310.2005 16687613

[B37] El Mansari M. Guiard B. P. Chernoloz O. Ghanbari R. Katz N. Blier P. (2010). Relevance of norepinephrine-dopamine interactions in the treatment of major depressive disorder. *CNS Neurosci. Ther.* 16 e1–e17 10.1111/j.1755-5949.2010.00146.x 20406250 PMC2904493

[B38] Foote S. L. Aston-Jones G. Bloom F. E. (1980). Impulse activity of locus coeruleus neurons in awake rats and monkeys is a function of sensory stimulation and arousal. *Proc. Natl. Acad. Sci. U.S.A.* 77 3033–3037 10.1073/pnas.77.5.3033 6771765 PMC349541

[B39] Gao W. J. Goldman-Rakic P. S. (2003). Selective modulation of excitatory and inhibitory microcircuits by dopamine. *Proc. Natl. Acad. Sci. U.S.A.* 100 2836–2841 10.1073/pnas.262796399 12591942 PMC151427

[B40] Gao W. J. Krimer L. S. Goldman-Rakic P. S. (2001). Presynaptic regulation of recurrent excitation by D1 receptors in prefrontal circuits. *Proc. Natl. Acad. Sci. U.S.A.* 98 295–300 10.1073/pnas.98.1.295 11134520 PMC14584

[B41] Gao W. J. Wang Y. Goldman-Rakic P. S. (2003). Dopamine modulation of perisomatic and peridendritic inhibition in prefrontal cortex. *J. Neurosci.* 23 1622–1630 12629166 10.1523/JNEUROSCI.23-05-01622.2003 PMC6741986

[B42] Gesi M. Soldani P. Giorgi F. S. (2000). A. Santinami, I. Bonaccorsi, and F. Fornai, The role of the locus coeruleus in the development of Parkinson’s disease. *Neurosci. Biobehav. Rev.* 24 655–668 10.1016/S0149-7634(00)00028-2 10940440

[B43] Goldman-Rakic P. S. (1994). Working memory dysfunction in schizophrenia. *J. Neuropsychiatry Clin. Neurosci.* 6 348–357 7841806 10.1176/jnp.6.4.348

[B44] Goldman-Rakic P. S. Selemon L. D. (1997). Functional and anatomical aspects of prefrontal pathology in schizophrenia. *Schizophr. Bull.* 23 437–458 10.1093/schbul/23.3.437 9327508

[B45] Goldman-Rakic P. S. Castner S. A. Svensson T. H. Siever L. J. Williams G. V. (2004). Targeting the dopamine D1 receptor in schizophrenia: insights for cognitive dysfunction. *Psychopharmacology* 174 3–16 10.1007/s00213-004-1793-y 15118803

[B46] Grace A. A. Floresco S. B. Goto Y. Lodge D. J. (2007). Regulation of firing of dopaminergic neurons and control of goal-directed behaviors. *Trends Neurosci.* 30 220–227 10.1016/j.tins.2007.03.003 17400299

[B47] Grenhoff J. Nisell M. Ferre S. Aston-Jones G. Svensson T. H. (1993). Noradrenergic modulation of midbrain dopamine cell firing elicited by stimulation of the locus coeruleus in the rat. *J. Neural Transm. Gen. Sect.* 93 11–25 10.1007/BF01244934 8373553

[B48] Grimm J. Mueller A. Hefti F. Rosenthal A. (2004). Molecular basis for catecholaminergic neuron diversity. *Proc. Natl. Acad. Sci. U.S.A.* 101 13891–13896 10.1073/pnas.0405340101 15353588 PMC518849

[B49] Grzanna R. Molliver M. E. (1980). The locus coeruleus in the rat: an immunohistochemical delineation. *Neuroscience* 5 21–40 10.1016/0306-4522(80)90068-8 6988734

[B50] Haber S. N. Fudge J. L. (1997). The primate substantia nigra and VTA: integrative circuitry and function. *Crit. Rev. Neurobiol.* 11 323–342 10.1615/CritRevNeurobiol.v11.i4.40 9336716

[B51] Horvitz J. C. (2000). Mesolimbocortical and nigrostriatal dopamine responses to salient non-reward events. *Neuroscience* 96 651–656 10.1016/S0306-4522(00)00019-1 10727783

[B52] Lammel S. Hetzel A. Hackel O. Jones I. Liss B. Roeper J. (2008). Unique properties of mesoprefrontal neurons within a dual mesocorticolimbic dopamine system. *Neuron* 57 760–773 10.1016/j.neuron.2008.01.022 18341995

[B53] Lammel S. Lim B. K. Malenka R. C. (2013). Reward and aversion in a heterogeneous midbrain dopamine system. *Neuropharmacology.* 46(Part B) 351–359 10.1016/j.neuropharm.2013.03.019 PMC3778102 23578393

[B54] Lammel S. Lim B. K. Ran C. Huang K. W. Betley M. J. Tye K. M. (2012). Input-specific control of reward and aversion in the ventral tegmental area. *Nature* 491 212–217 10.1038/nature11527 23064228 PMC3493743

[B55] Lategan A. J. Marien M. R. Colpaert F. C. (1990). Effects of locus coeruleus lesions on the release of endogenous dopamine in the rat nucleus accumbens and caudate nucleus as determined by intracerebral microdialysis. *Brain Res*. 523 134–138 10.1016/0006-8993(90)91646-X 1698514

[B56] Lewis D. A. Campbell M. J. Foote S. L. Goldstein M. Morrison J. H. (1987). The distribution of tyrosine hydroxylase-immunoreactive fibers in primate neocortex is widespread but regionally specific. *J. Neurosci.* 7 279–290 2879896 10.1523/JNEUROSCI.07-01-00279.1987 PMC6568855

[B57] Loughlin S. E. Foote S. L. Bloom F. E. (1986a). Efferent projections of nucleus locus coeruleus: topographic organization of cells of origin demonstrated by three-dimensional reconstruction. *Neuroscience* 18 291–306 10.1016/0306-4522(86)90155-7 3736860

[B58] Loughlin S. E. Foote S. L. Grzanna R. (1986b). Efferent projections of nucleus locus coeruleus: morphologic subpopulations have different efferent targets. *Neuroscience* 18 307–319 10.1016/0306-4522(86)90156-9 3736861

[B59] Loughlin S. E. Foote S. L. Fallon J. H. (1982). Locus coeruleus projections to cortex: topography, morphology and collateralization. *Brain Res. Bull*. 9 287–294 10.1016/0361-9230(82)90142-3 7172032

[B60] Mao Z. M. Arnsten A. F. Li B. M. (1999). Local infusion of an alpha-1 adrenergic agonist into the prefrontal cortex impairs spatial working memory performance in monkeys. *Biol. Psychiatry* 46 1259–1265 10.1016/S0006-3223(99)00139-0 10560031

[B61] McGaughy J. Newman L. A. Darling J. (2008a). Atomoxetine reverses attentional deficits produced by noradrenergic deafferentation of medial prefrontal cortex. *Psychopharmacology* 200 39–50 10.1007/s00213-008-1097-8 18568443 PMC10719959

[B62] McGaughy J. Ross R. S. Eichenbaum H. (2008b). Noradrenergic, but not cholinergic, deafferentation of prefrontal cortex impairs attentional set-shifting. *Neuroscience* 153 63–71 10.1016/j.neuroscience.2008.01.064 18355972 PMC2615225

[B63] McMillan P. J. White S. S. Franklin A. Greenup J. L. Leverenz J. B. Raskind M. A. (2011). Differential response of the central noradrenergic nervous system to the loss of locus coeruleus neurons in Parkinson’s disease and Alzheimer’s disease. *Brain Res*. 1373 240–252 10.1016/j.brainres.2010.12.015 21147074 PMC3038670

[B64] Miguelez C. Grandoso L. Ugedo L. (2011). Locus coeruleus and dorsal raphe neuron activity and response to acute antidepressant administration in a rat model of Parkinson’s disease. *Int. J. Neuropsychopharmacol*. 14 187–200 10.1017/S146114571000043X 20426885

[B65] Mingote S. de Bruin J. P. Feenstra M. G. (2004). Noradrenaline and dopamine efflux in the prefrontal cortex in relation to appetitive classical conditioning. *J. Neurosci.* 24 2475–2480 10.1523/JNEUROSCI.4547-03.2004 15014123 PMC6729496

[B66] Morilak D. A. Barrera G. Echevarria D. J. Garcia A. S. Hernandez A. Ma S. (2005). Role of brain norepinephrine in the behavioral response to stress. *Prog. Neuropsychopharmacol. Biol. Psychiatry* 29 1214–1224 10.1016/j.pnpbp.2005.08.007 16226365

[B67] Morrison J. H. Grzanna R. Molliver M. E. Coyle J. T. (1978). The distribution and orientation of noradrenergic fibers in neocortex of the rat: an immunofluorescence study. *J. Comp. Neurol.* 181 17–39 10.1002/cne.901810103 355267

[B68] Newman L. A. Darling J. McGaughy J. (2008). Atomoxetine reverses attentional deficits produced by noradrenergic deafferentation of medial prefrontal cortex. *Psychopharmacology* (*Berl.*) 200 39–50 10.1007/s00213-008-1097-8 18568443 PMC10719959

[B69] Porrino L. J. Goldman-Rakic P. S. (1982). Brainstem innervation of prefrontal and anterior cingulate cortex in the rhesus monkey revealed by retrograde transport of HRP. *J. Comp. Neurol.* 205 63–76 10.1002/cne.902050107 6121826

[B70] Rajkowski J. Kubiak P. Aston-Jones G. (1994). Locus coeruleus activity in monkey: phasic and tonic changes are associated with altered vigilance. *Brain Res. Bull*. 35 607–616 10.1016/0361-9230(94)90175-9 7859118

[B71] Robbins T. W. Arnsten A. F. (2009). The neuropsychopharmacology of fronto-executive function: monoaminergic modulation. *Annu. Rev. Neurosci.* 32 267–287 10.1146/annurev.neuro.051508.135535 19555290 PMC2863127

[B72] Robertson S. D. Plummer N. W. de Marchena J. Jensen P. (2013). Developmental origins of central norepinephrine neuron diversity. *Nat. Neurosci*. 16 1016–1023 10.1038/nn.3458 23852112 PMC4319358

[B73] Roeper J. (2013). Dissecting the diversity of midbrain dopamine neurons. *Trends Neurosci*. 36 336–342 10.1016/j.tins.2013.03.003 23582338

[B74] Rossetti Z. L. Carboni S. (2005). Noradrenaline and dopamine elevations in the rat prefrontal cortex in spatial working memory. *J. Neurosci.* 25 2322–2329 10.1523/JNEUROSCI.3038-04.2005 15745958 PMC6726105

[B75] Sara S. J. Bouret S. (2012). Orienting and reorienting: the locus coeruleus mediates cognition through arousal. *Neuron* 76 130–141 10.1016/j.neuron.2012.09.011 23040811

[B76] Schultz W. (2007). Multiple dopamine functions at different time courses. *Annu. Rev. Neurosci.* 30 259–288 10.1146/annurev.neuro.28.061604.135722 17600522

[B77] Seamans J. K. Yang C. R. (2004). The principal features and mechanisms of dopamine modulation in the prefrontal cortex. *Prog. Neurobiol.* 74 1–58 10.1016/j.pneurobio.2004.05.006 15381316

[B78] Seu E. Lang A. Rivera R. J. Jentsch J. D. (2009). Inhibition of the norepinephrine transporter improves behavioral flexibility in rats and monkeys. *Psychopharmacology* (*Berl.*) 202 505–519 10.1007/s00213-008-1250-4 18604598 PMC2634830

[B79] Snyder K. Wang W. W. Han R. McFadden K. Valentino R. J. (2012). Corticotropin-releasing factor in the norepinephrine nucleus, locus coeruleus, facilitates behavioral flexibility. *Neuropsychopharmacology* 37 520–530 10.1038/npp.2011.218 21993205 PMC3242313

[B80] Stevens A. A. Goldman-Rakic P. S. Gore J. C. Fulbright R. K. Wexler B. E. (1998). Cortical dysfunction in schizophrenia during auditory word and tone working memory demonstrated by functional magnetic resonance imaging. *Arch. Gen. Psychiatry* 55 1097–1103 10.1001/archpsyc.55.12.1097 9862553

[B81] Stuber G. D. Klanker M. de Ridder B. Bowers M. S. Joosten R. N. Feenstra M. G. (2008). Reward-predictive cues enhance excitatory synaptic strength onto midbrain dopamine neurons. *Science* 321 1690–1692 10.1126/science.1160873 18802002 PMC2613864

[B82] Swanson L. W. (1982). The projections of the ventral tegmental area and adjacent regions: a combined fluorescent retrograde tracer and immunofluorescence study in the rat. *Brain Res. Bull.* 9 321–353 10.1016/0361-9230(82)90145-9 6816390

[B83] Szot P. Miguelez C. White S. S. Franklin A. Sikkema C. Wilkinson C. W. (2010). A comprehensive analysis of the effect of DSP4 on the locus coeruleus noradrenergic system in the rat. *Neuroscience* 166 279–291 10.1016/j.neuroscience.2009.12.027 20045445 PMC4060967

[B84] Ungless M. A. Grace A. A. (2012). Are you or aren t you? Challenges associated with physiologically identifying dopamine neurons. *Trends Neurosci.* 35 422–430 10.1016/j.tins.2012.02.003 22459161 PMC3383926

[B85] Valentino R. J. Foote S. L. (1987). Corticotropin-releasing factor disrupts sensory responses of brain noradrenergic neurons. *Neuroendocrinology* 45 28–36 10.1159/000124700 3492683

[B86] Valentino R. J. Foote S. L. (1988). Corticotropin-releasing hormone increases tonic but not sensory-evoked activity of noradrenergic locus coeruleus neurons in unanesthetized rats. *J. Neurosci.* 8 1016–1025 3258021 10.1523/JNEUROSCI.08-03-01016.1988 PMC6569228

[B87] Volkow N. D. Wang G. J. Fowler J. S. Tomasi D. (2012). Addiction circuitry in the human brain. *Annu. Rev. Pharmacol. Toxicol.* 52 321–336 10.1146/annurev-pharmtox-010611-134625 21961707 PMC3477468

[B88] Volkow N. D. Wang G. J. Fowler J. S. Tomasi D. Telang F. (2011). Addiction: beyond dopamine reward circuitry. *Proc. Natl. Acad. Sci. U.S.A.* 108 15037–15042 10.1073/pnas.1010654108 21402948 PMC3174598

[B89] Wang H. X. Waterhouse B. D. Gao W. J. (2013). Selective suppression of excitatory synapses on GABAergic interneurons by norepinephrine in juvenile rat prefrontal cortical microcircuitry. *Neuroscience* 246 312–328 10.1016/j.neuroscience.2013.05.009 23684615 PMC3697018

[B90] Weinshenker D. (2008). Functional consequences of locus coeruleus degeneration in Alzheimer’s disease. *Curr. Alzheimer Res.* 5 342–345 10.2174/156720508784533286 18537547

[B91] Williams G. V. Goldman-Rakic P. S. (1995). Modulation of memory fields by dopamine D1 receptors in prefrontal cortex. *Nature* 376 572–575 10.1038/376572a0 7637804

